# Electrophysiological indicators of surprise and entropy in dynamic task-switching environments

**DOI:** 10.3389/fnhum.2013.00300

**Published:** 2013-07-03

**Authors:** Bruno Kopp, Florian Lange

**Affiliations:** ^1^Department of Neurology, Hannover Medical SchoolHannover, Germany; ^2^Cognitive Neurology, Technische Universität BraunschweigBraunschweig, Germany; ^3^Department of Research Methods and Biopsychology, Technische Universität BraunschweigBraunschweig, Germany

**Keywords:** event-related potentials, P3b, P3a, task-switching, uncertainty, surprise, entropy, orienting response

## Abstract

This event-related brain potential (ERP) study aimed at bridging two hitherto widely separated domains of cognitive neuroscience. Specifically, we combined the analysis of cognitive control in a cued task-switching paradigm with the fundamental question of how uncertainty is encoded in the brain. Two functional models of P3 amplitude variation in cued task-switching paradigms were put to an empirical test: (1) According to the P3b surprise hypothesis, parietal P3b waveforms are related to surprise over switch cues. (2) According to the P3a entropy hypothesis, frontal P3a waveforms are associated with entropy over switch outcomes. In order to examine these hypotheses, we measured the EEG while sixteen healthy young participants performed cued task-switching paradigms closely modeled to the Wisconsin Card Sorting Test (WCST). We applied a factorial design, with number of tasks (two vs. three viable tasks), cue explicitness (task cuing vs. transition cuing), and cue contingency (prospectively-signaled cuing vs. feedback-based cuing) as independent variables. The ERP results replicated the commonly reported P3b effect associated with task switches, and further showed that P3a amplitudes were related to the entropy of switch outcomes, thereby supporting both hypotheses. Based on these ERP data, we suggest that surprise over task switches, and entropy over switch outcomes, constitute dissociable functional correlates of P3b and P3a ERP components in task-switching paradigms, respectively. Finally, a theoretical integration of the findings is proposed within the framework of Sokolov's (1966) entropy model of the orienting response (OR).

## Introduction

In task-switching paradigms, one of several viable stimulus-response mappings (also referred to as task sets) needs to be executed at any one time, based on contextual information provided by instructions or by other stimuli (*cued* task-switching; see Monsell, 2003; Kiesel et al., 2010; for reviews). Task-switching paradigms thus provide dynamic environments which involve frequent state transitions, and which thereby probe contextual adjustment by the performers. Task-switching paradigms are often considered benchmark in research on neurocognitive mechanisms of executive control (Shallice et al., 2008; Nyhus and Barceló, 2009; Kopp and Wessel, 2011) since deficient contextual adjustment represents one of the hallmarks of executive dysfunctions which occur in some brain-lesioned patients (Kopp, 2012).

In principle, there exist two categories of models of cognitive control in task-switching paradigms, which we here refer to as meta-level models and task-level models (see Mayr et al., 2013, for a more detailed discussion). Meta-level models assume that a task switch “… necessitates a process (or a set of processes) that operates on an abstract, hierarchically higher level than that of specific tasks” (Mayr et al., 2013; p. 491). However, Kiesel et al. (2010) concluded that the necessity of higher-level control has not yet been settled in the relevant literature. Task-level models assume that task control can be established exclusively through mutual competition between task sets, without any requirement of higher-level processes which should be related to the need to switch back and forth between task sets (e.g., Gilbert and Shallice, 2002; Logan and Bundesen, 2003).

**Figure 1:**
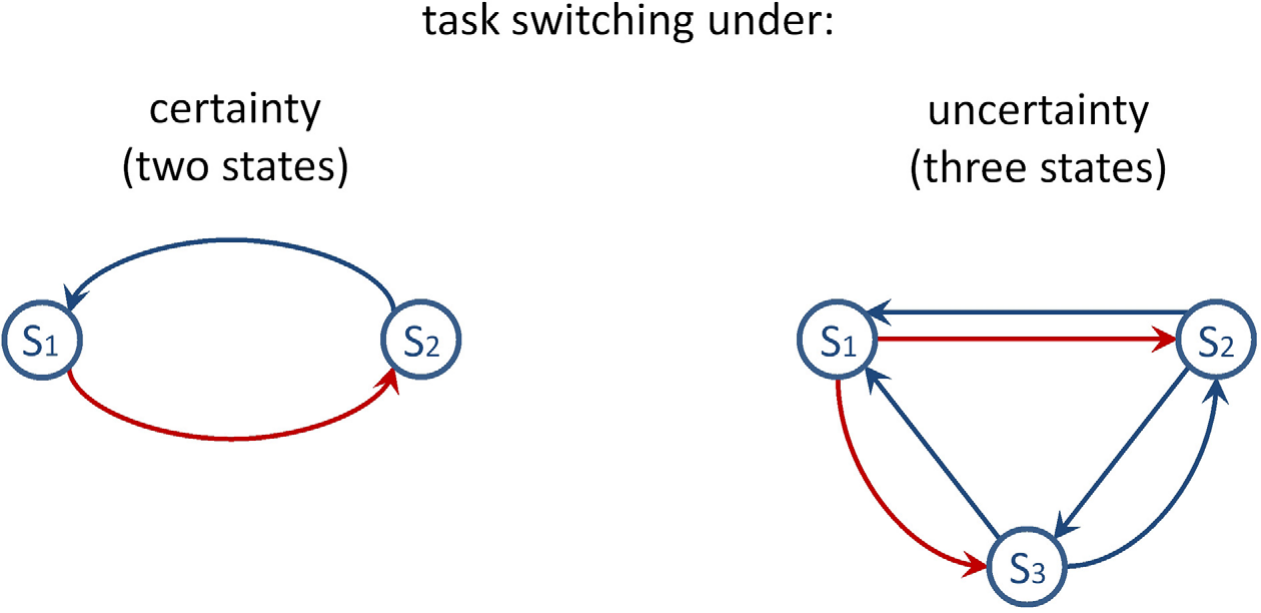
**Task-switching paradigms considered as dynamic environments which involve frequent state transitions. Left panel:** The simple diagram shows state transitions when two tasks are viable. The red arrow indicates that S1 passes on to S2 (i.e., switching between task sets consists of a one-alternative choice). The blue arrow illustrates the alternative switch between task sets. **Right panel:** The diagram shows state transitions when three tasks are viable. The red arrows indicate that S1 passes on to S2 or to S3 (i.e., switching between task sets consists of a two-alternative choice). The blue arrows illustrate possible other task switches.

Conceptualizing meta-level task control as an all-or-none phenomenon may be an inadequate oversimplification of a continuously variable phenomenon. For example, the dynamics of task control might necessitate the contribution of meta-level processes to the degree that decisions about task sets are uncertain (Kepecs and Mainen, 2012). Figure 1 depicts two state transition diagrams which illustrate task switching under certainty and under uncertainty. Specifically, inspection of Figure 1 reveals that transitions between task sets can be conducted with certainty on two-task paradigms, whereas three-task paradigms imply uncertain transitions between task sets. This consideration led to the present study which focuses on task-set uncertainty. Our study thereby touches the fundamental question of how uncertainty influences behavior and how it is encoded in the brain (Bach and Dolan, 2012; Badre et al., 2012).

The Wisconsin Card Sorting Test (WCST; Berg, 1948; Grant and Berg, 1948; Heaton et al., 1993) represents a clinical benchmark task-switching paradigm (Milner, 1963; Nyhus and Barceló, 2009; Kopp and Wessel, 2011), since patients with frontal brain damage commit more errors on the WCST than patients with non-frontal damage (Barceló and Knight, 2002; Demakis, 2003). The WCST possesses two highly specific, yet important characteristics: (1) WCST stimuli differ with regard to the color, shape, and number of depicted objects, and these three stimulus dimensions define *three* viable tasks. (2) Further, to cite the original articles: “As the S [subject] sorted the response cards he was informed whether he was ‘right’ or ‘wrong’.” (Berg, 1948, p. 16; Grant and Berg, 1948, p. 405). Thus, solely “wrong”-cues, following erroneous sorts, signal the need to switch task sets, but they do *not* indicate the currently prevailing task on the WCST. Taken together, there are at least three hypothetical explanations for why the WCST is found to be one of the few task-switching paradigms with documented sensitivity for frontal lobe dysfunction (Barceló and Knight, 2002; Demakis, 2003). (1) The WCST may place high load on working memory (the *memory load* hypothesis). (2) Successful performance on the WCST may hinge on efficient action-outcome monitoring (the *contingency* hypothesis). (3) The WCST may challenge task switching under uncertainty (the *uncertainty* hypothesis). The rationale of each of these three hypotheses will be briefly discussed in the following paragraphs.

The memory load hypothesis rests on the fact that contextual cues on the WCST are *transition* cues, indicating that task sets need to be changed, without specifying which of the three tasks is currently effective, whereas *task* cues would provide explicit information about the currently effective task. Schneider and Logan (2007) suggested that transition cues place greater demands on the retrieval of task sets in comparison to task cues. Forstmann et al. (2005) showed functional activation of lateral prefrontal areas by transition cues, but not by task cues. Further, based on event-related brain potential (ERP) recordings, West et al. (2011) reported that transition cues evoked more pronounced frontal positivities than task cues did. This analysis led to one of our experimental manipulations (*cue explicitness*) in which labels such as “switch” (rather than “wrong” as in the original WCST) and “repeat” (rather than “right” as in the original WCST) were used for transition-cuing, whereas the labels “color,” “shape,” and “number” constituted the stimulus material for explicit task cuing.

The contingency hypothesis focuses on the fact that contextual cuing rests on feedback stimuli on the WCST, such that successful performance requires efficient action-outcome monitoring (Kopp and Wessel, 2011). Action-outcome prediction (Alexander and Brown, 2010, 2011) has been considered to form the basis of performance monitoring, a widely studied frontal function (Gehring and Knight, 2000; Ullsperger et al., 2002; Ridderinkhof et al., 2004; Wessel et al., 2012). Throughout this article, we call the feedback-based method of contextual cuing *feedback-based* (*F-B*) cuing. In contrast to F-B cuing, many task-switching paradigms provide information about the currently effective task by delivering contextual cues before the appearance of the imperative stimuli. Throughout this article, this contextual cuing method will be called *prospectively-signaled* (*P-S*) cuing. This analysis led to another experimental manipulation (*cue contingency*), in which we compared the effects of F-B cues with the effects of P-S cues, as further described below.

**Figure 2:**
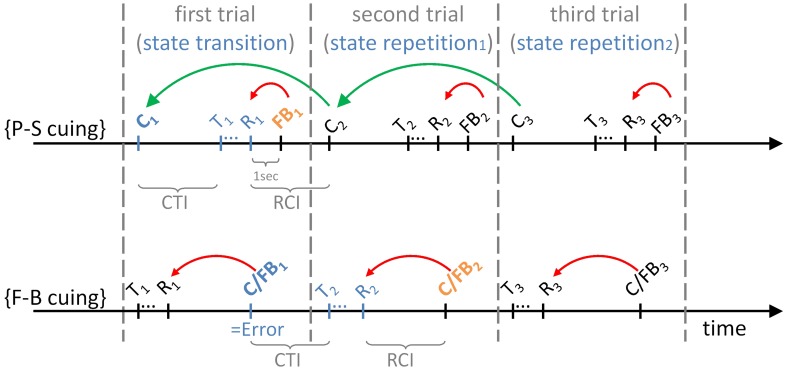
**The temporal sequence of events on P-S cuing and on F-B cuing conditions.** The figure illustrates the initial three trials of task runs. *C*_*x*_, task cue on trial *x*{*x* = 1 … 3}; FB_*x*_, feedback stimulus on trial *x*{*x* = 1 … 3}; C/FB_*x*_, hybrid cue/feedback stimulus on trial *x*{*x* = 1 … 3}; T_*x*_, target stimulus on trial *x*{*x* = 1 … 3}; R_*x*_, response on trial *x*{*x* = 1 … 3}. CTI, cue-target interval (3 s); RCI, response-cue interval (3 s). Curved arrows indicate the contingencies: C_*x*_-cues inform about possible state transitions, i.e., the currently effective task relative to the task that was effective on the previous trial on the P-S cuing condition; additional FB_*x*_-feedback stimuli inform about the correctness of the response, R_*x*_, on the P-S cuing condition. Specifically, C_1_-cues (shown in blue) require set shifts, and FB_1_-feedback stimuli (shown in orange) enable evaluating the task-set decision. C/FB_*x*_-hybrid stimuli inform about the correctness of the response, R_*x*_, on the F-B cuing condition. In the absence of other information, this feedback simultaneously serves as contextual information about possible state transitions. Specifically, a set shift is required when C/FB_1_-stimuli (shown in blue) signal that R_1_ was erroneous, whereas C/FB_2_-stimuli (shown in orange) enable evaluating the task-set decision.

Figure 2 illustrates event structures over time within the initial three trials of task runs, separately for P-S cuing and for F-B cuing. It can be seen that cues (e.g., *C*_1_; read: “*cue* on trial *1*”) precede targets (e.g., *T*_1_; read: “*target* on trial *1*”) on P-S cuing conditions, such that *R*_1_ (read: “*response* on trial *1*”) will be correct (in the “ideal” performer). In contrast, because targets (e.g., *T*_1_) precede cue/feedback events (e.g., *C*/*FB*_1_; read: “*cue/feedback* on trial *1*”) on F-B cuing conditions, *R*_1_ will necessarily be incorrect (even in the “ideal” performer). As a corollary, while first trials are state-transition trials on both cuing conditions, set shifts are expected to occur on first trials on P-S cuing conditions, whereas set shifts are expected to occur on second trials on F-B cuing conditions (see Table 1).

**Table 1:** **The table shows the initial three trials of task runs for clarification of our nomenclature**.

	**First trial**	**Second trial**	**Third trial**
	State transition	State repetition_1_	State repetition_2_
P-S cuing	Set shift	First set repetition	Set repetition
F-B cuing	Set repetition (error)	Set shift	First set repetition

Set shifts, but not state transitions, are distributed across different trials on P-S cuing and F-B cuing conditions.

Further, the green curved arrows in Figure 2 indicate that P-S cues inform about the occurrence of state transitions vs. repetitions, irrespective of performance accuracy. Feedback information about performance accuracy is solely provided by additional feedback stimuli in P-S cuing paradigms (red curved arrows). Thus, task cues and feedback stimuli are dispersed in time within each trial on P-S cuing conditions. This characteristic of P-S cuing differs from F-B cuing where stimuli concomitantly serve as task cues and feedback stimuli (hence the notation *C/FB*, indicating that these events are hybrid *cue* and *feedback* events; red curved arrows).

The simplest way to evaluate the uncertainty hypothesis is to manipulate the number of viable tasks (cf. Figure 1). Subjects need to handle three viable task sets on the WCST, (i.e., to inhibit the previous set, e.g., “shape,” and to consider the other two sets for responding, e.g., “color” and “number”), whereas the use of two tasks is common in many task-switching experiments (Kiesel et al., 2010). Thus, as illustrated in Figure 1, whereas the WCST incorporates two-alternative choice (2AC) task-set decisions, many task-switching paradigms demand one-alternative choice (1AC) task-set decisions (i.e., to inhibit the previous set, e.g., “shape,” and to consider the other set for responding, e.g., “color,” when “number” is not a viable alternative). Note further that explicit task cuing generally implies 1AC task-set decisions, irrespective of the number of viable rules, because explicit task cues (e.g., “color”) specify the correct task directly.

The factorial combination of cue explicitness (task cuing vs. transition cuing), cue contingency (P-S vs. F-B cuing), and number of tasks (two vs. three tasks) led to differential dynamics of task-set decisions, as shown in Table 2. Decisions (*D*) to switch task sets follow *C*_1_, and the presentation of *FB*_1_ (read: “*feedback* on trial *1*”) allows to evaluate (*E*) their correctness within the first trial on P-S cuing conditions. In contrast, decisions to switch sets task sets follow *C*/*FB*_1_ (which indicates erroneous performance), and evaluating their correctness is not possible before *C*/*FB*_2_ on the second trial on F-B cuing conditions. Table 2 offers an overview over the various possibilities, basically showing the occurrence of 1AC task-set decisions, and their evaluation, on all explicit task-cuing conditions (i.e., irrespective of the number of possible tasks) as well as on transition-cuing conditions with only two viable tasks. The sole exception from this pattern emerges on transition-cuing conditions with three viable tasks, where 2AC task-set decisions, and their evaluation, are required. Thus, 2AC task-set decisions are required on three-task transition-cuing conditions (task-switching under uncertainty), whereas 1AC task-set decisions are sufficient on all remaining conditions (task switching under certainty).

**Table 2:** **Task switching under certainty/uncertainty across the initial three trials of task runs**.

**P-S cuing**	**C_1_**	**FB_1_**	**C_2_**	**FB_2_**	**C_3_**	**FB_3_**
Task (2, 3 tasks)	D:1AC	E:1AC				
Transition (2 tasks)	D:1AC	E:1AC				
Transition (3 tasks)	D:2AC	E:2AC		(E:1AC)		
**F-B cuing**		**C/FB_1_**		**C/FB_2_**		**C/FB_3_**
Task (2, 3 tasks)		D:1AC		E:1AC		
Transition (2 tasks)		D:1AC		E:1AC		
Transition (3 tasks)		D:2AC		E:2AC		(E:1AC)

*C_x_, task cue on trial x {x = 1 … 3}; FB_x_, feedback stimulus on trial x {x = 1 … 3}; C/FB_x_, hybrid cue/feedback stimulus on trial x {x = 1 … 3}; task (2,3 tasks), two- and three-task task cuing; transition (2 tasks), two-transition cuing; transition (3 tasks), three-task transition cuing; D:, task-set decision; E:, decision evaluation; 1AC, one-alternative choice; 2AC, two-alternative choice. Note that 2AC-decisions (D:2AC) occur exclusively on three-task transition cuing conditions. These uncertain task decisions can be evaluated (E:2AC) through FB_1_ on P-S cuing conditions and through C/FB_2_ on F-B cuing conditions. The bracketed terms [i.e., (E:1AC)] indicate that an additional set switch (of the D:1AC-type) is required in case that FB_1_ on P-S cuing conditions and C/FB_2_ on F-B cuing conditions reveal incorrect initial task-set choices*.

**Figure 3:**
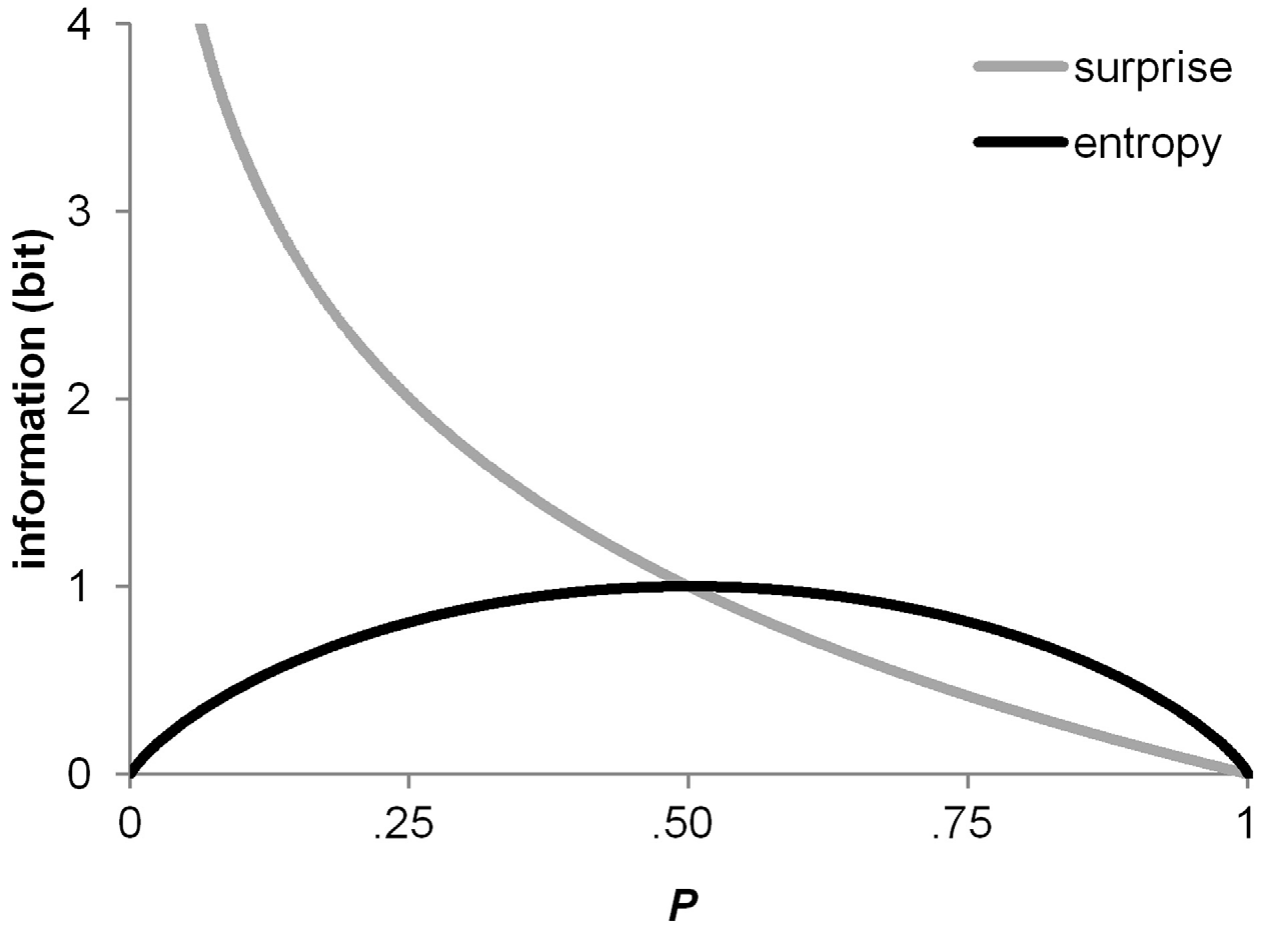
**The surprise function and the entropy function as a function of probability over a binary random variable**.

In the remainder of our introduction, we apply two classical measures of uncertainty, i.e., surprise and entropy, to task-switching. Specifically, we formalize surprise over switch cues and entropy over negative outcomes in order to prepare the derivation of hypotheses about the functional significance of two ERP measures which are consistently found in task-switching paradigms. To begin with, it is well-known that surprise, *I(X)*,

(1) $$I(X=i)=-(\log_{2}P_{i})$$

and entropy, *H(X)*,

(2) $$H(X=i)=-(P_{i}\log_{2}P_{i}+(1-P_{i})\log_{2}(1-P_{i}))$$

for a binary random variable *X* represent two different measures of uncertainty (Shannon and Weaver, 1948). Figure 3 shows surprise and entropy as a function of probability for a binary random variable *X* [as in (1), (2)]. Note that the binary entropy function reaches a maximum at equal probabilities (i.e., at *P* = 0.5), whereas maximum surprise is associated with rareness or improbability (i.e., when *P* → 0.0).

Let

(3) $$\begin{array}{l} P_{s}(n)=P(t(n)=s|t(n-1))with\;s\;\epsilon \{s\;(switch),\\ \;ns(no-switch)\};P_{ns}=1-P_{s} \end{array}$$

denote a *subjective probability estimate* that a state transition will occur on trial *n* ϵ {1, …, *N*}, given a sequence *t*(*n* − 1) = *t*(1), *t*(2), …, *t*(*n* − 1) of *n* − 1 former trials. Note that trial *t(n)* in (3) has not yet been observed, therefore, a subjective probability distribution *P*_*s*_(*n*) for *all* possibilities {*s*, *ns*} on *t(n)* is of interest. However, once *s* has been observed on *t(n)* (which is only a *single* value *s* out of set {*s*, *ns*}), the respective subjective probability *P*_*s*_(*n*) can be used to calculate the degree of *surprise* (Shannon and Weaver, 1948; Kolossa et al., 2013)

(4) $$I(n)=-\log_{2}P_{s}(n).$$

Let

(5) $$\begin{array}{l} P_{w}(n)=P(t(n)=p|t(n-1))with\;w\;\epsilon \{w\;(wrong),\\ \;r(right)\;outcome\};P_{r}=1-P_{w} \end{array}$$

denote a *subjective probability estimate* that a “wrong”-outcome will occur on trial *t(n)*, given a sequence of *n* − 1 former trials. Note that the outcome on *t(n)* has not yet been observed, therefore, a subjective probability distribution *P*_*w*_(*n*) for all possibilities *{w, r}* on *t(n)* is of interest. However, once *w* has been observed on *t(n)*, the respective subjective probability *P*_*w*_(*n*) can be used to calculate the degree of *entropy* (Shannon and Weaver, 1948; Kolossa et al., 2013)

(6) $$H(n)=-(P_{w}(n)\log_{2}P_{w}(n)+P_{r}(n)\log_{2}P_{r}(n)).$$

**Figure 4:**
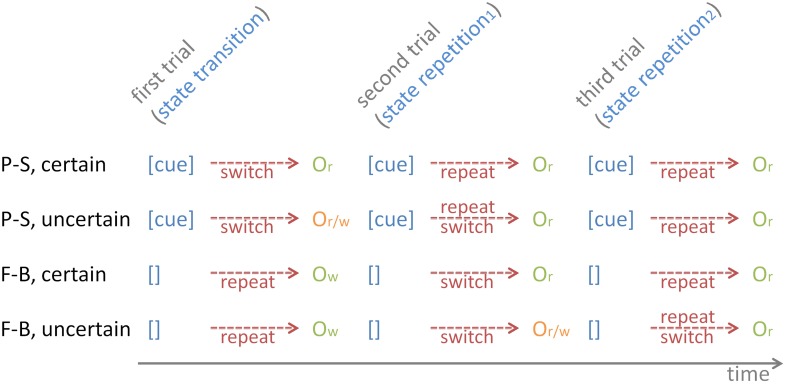
**The temporal sequence of contextual cues and outcome stimuli on P-S cuing and on F-B cuing conditions, separately for certain and uncertain conditions.** The figure illustrates the initial three trials of task runs. Contextual cues are provided on P-S cuing, but not on F-B cuing (depicted as []), which leads to erroneous task-set repetitions on state-transition trials, and concomitantly to “wrong”-outcomes (O_*w*_). Note the uncertainty of outcomes (“right” or “wrong,” O_*r*/_*w*) on uncertain P-S state-transition trials and on uncertain F-B state-repetition_1_ trials. Depending on the outcome valence, task-set repetitions or task-set switches are required on subsequent trials.

Figure 4 illustrates that the *subjective probability estimates P*_*w*_(*n*) need to be further conditioned on the serial position of *t(n)* within task runs. There are only two instances where *P*_*w*_(*n*) will deviate from either *P* = 0.0 or *P* = 1.0, i.e., on the first trial on uncertain P-S cuing conditions, and on the second trial on uncertain F-B cuing conditions. Table 3 presents these conditional probabilities *P*_*w*_(*n*) as they result in an ideal performer.

**Table 3:** **Conditional probabilities for negative trial outcomes in an ideal performer, separately for P-S cuing and F-B cuing as well as for certain and uncertain cuing conditions, further conditioned on the serial position of trials within task runs**.

	**First trial**	**Second trial**	**Third trial**
	**State transition**	**State repetition_1_**	**State repetition_2_**
P-S cuing, certain	*P*_*w*_ = 0.0	*P*_*w*_ = 0.0	*P*_*w*_ = 0.0
P-S cuing, uncertain	*P*_*w*_ = 0.5	*P*_*w*_ = 0.0	*P*_*w*_ = 0.0
F-B cuing, certain	*P*_*w*_ = 1.0	*P*_*w*_ = 0.0	*P*_*w*_ = 0.0
F-B cuing, uncertain	*P*_*w*_ = 1.0	*P*_*w*_ = 0.5	*P*_*w*_ = 0.0

The current study made use of ERP measures to examine hypothetical explanations for the documented frontal lobe sensitivity of the WCST. We now briefly discuss the P3b and P3a variants of the P300 ERP component (see for Polich, 2007; Duncan et al., 2009, for reviews) which are associated with uncertainty (see Kopp, 2008, for a conceptual review) and which play major roles in WCST-like task switching (Barceló, 2003). Barceló et al. (2002) used a modified version of the WCST and analyzed ERPs to the cue/feedback tones which were presented after every trial. ERPs to switch cues/feedbacks showed large frontal positivities (350–400 ms) as well as large later parietal positivities (550–600 ms) which decreased in amplitude across task runs. These ERP effects were interpreted as modulation of parietal P3b and frontal P3a waveforms as a result of switching task sets.

The observation of switch-related enhancements of parietal P3b amplitudes is a ubiquitous finding in the task-switching literature (Rushworth et al., 2002, 2005; Karayanidis et al., 2003, 2010; Kieffaber and Hetrick, 2005; Nicholson et al., 2005; Astle et al., 2006, 2008; Swainson et al., 2006; Jost et al., 2008; Travers and West, 2008; Periáñez and Barceló, 2009; Wylie et al., 2009; Gajewski and Falkenstein, 2011). Further, it is well-recognized that “surprising events elicit a large P300 component” (Donchin, 1981, p. 498) as specified in Donchin's surprise hypothesis of P3b amplitude modulations (see also Kolossa et al., 2013). Applied to switch-related enhancements of parietal P3b amplitudes, cue-locked P3b amplitudes should be proportional to surprise *I*(*n*), i.e.,

(7) P3b(n)∝I(n)

with *I(n)* as defined in (4), i.e., the surprise over task-set switches.

Barceló et al. (2002) found a rapid decline of frontal P3a amplitudes across task runs which they interpreted as being related to task-set uncertainty. Note that prominent P3a waveforms in response to cue/feedback events were also found in other WCST-like task-switching studies (Kopp et al., 2005, 2006; Barceló et al., 2006; Cunillera et al., 2012). Barceló et al. (2006) put forward the hypothesis that modulations of P3a amplitudes are generally related to the entropy which is conveyed by eliciting stimuli. Applied to switch-related enhancements of frontal P3a amplitudes, feedback-locked P3a amplitudes should be proportional to entropy *H*(*n*), i.e.,

(8) P3a(n)∝H(n)

with *H(n)* as defined in (6) and in Table 3, i.e., the entropy over outcomes, conditioned on task-set (un-)certainty and serial position within task runs.

We started our analysis of WCST-like task switching with the formulation of three hypotheses. The memory load hypothesis led us to expect strong frontal activities which should be evoked by transition cues, as indicated by frontal P3a waveforms. In contrast, the contingency hypothesis led us to expect strong frontal activities which should be evoked by F-B cues, again as indicated by frontal P3a waveforms. Finally, the uncertainty hypothesis led to two predictions. First, surprise over task-set switches, being related to their rareness or improbability, should modulate the parietal P3b. Second, entropy over switch outcomes should modulate the frontal P3a, such that *P* = 0.5 outcomes should elicit enhanced P3a amplitudes in comparison to both, *P* = 1.0 and *P* = 0.0 outcomes, which should evoke less prominent P3a waveforms.

## Materials and methods

### Participants

A group of sixteen healthy undergraduate psychology students (*M* = 21.5 years; range 19–26 years; 14 females; 5 left-handed) participated for course credit. All had normal or corrected-to-normal vision. Nobody showed impairment in set switching abilities as indicated by the time they required to complete Trail Making Test A, 23.7 (*M*) ± 1.8 (*SE*) sec, and Trail Making Test B, 52.7 (*M*) ± 3.7 (*SE*) sec (Army Individual Test Battery, 1944).

The authors confirm that the research has been conducted according to all ethical standards imposed by their Ethics Committee at the Technische Universität Braunschweig, who approved the study. The study conforms to the Declaration of Helsinki. Written informed consent was obtained by all participants, according to the procedures imposed and approved by the above Ethics Committee.

### Stimulus materials and procedure

The experiment was controlled by the Presentation® software (Albany, CA; http://www.neurobs.com). Target displays consisted of four key cards per trial which appeared invariantly above one stimulus card, all configured around the center of a computer screen against white background (Eizo FlexScan T766 19″; Hakusan, Ishikawa, Japan; http://www.eizo.com/global). The stimulus arrangement subtended a visual angle of 4° horizontally and 2.5° vertically at a viewing distance of 1.5 m. Participants indicated their sorting choice by pressing one of four keys on a standard computer keyboard which were mapped to the spatial position of the key cards on the screen (“C,” left middle finger, equaling choice of the outside left key card; “V,” left index finger, equaling choice of inside left key card; “N,” right index finger, equaling choice of inside right key card; “M,” right middle finger, equaling choice of outside right key card). Target displays remained on screen until a response was recognized. We selected only those stimulus cards of the original 64-card version of the WCST which share one and only one attribute with each of the four key cards (i.e., the stimulus cards which match one key card by color, another keycard by shape, and a third key card by number). Twenty-four stimulus cards fulfill this criterion (Nelson, 1976).

One single sequence of trials was generated pseudo-randomly by one of the authors (Florian Lange). This particular sequence of trials was utilized repeatedly throughout all experimental conditions. Thus, the succession of stimulus cards was exactly identical for all experimental conditions. Each stimulus card was presented five times per condition, adding up to a total of 120 trials. The sequence of trials included 31 rule switches, yielding an average run length of 3.8 trials, with a range of three to five trials per run. The succession of runs was identical for all two-task experimental conditions such that these four conditions differed solely with regard to cue explicitness and cue contingency. Likewise, the succession of runs was identical for all three-task experimental conditions were the sequence of trials consisted of eleven runs of “color” and “shape” sorting, respectively, as well as ten runs of trials which required sorting according to the “number” of objects displayed on the stimulus cards.

Depending on the experimental condition, target stimuli were differentially surrounded in time by cue and feedback stimuli (see Figure 2). On P-S cuing conditions, cues (C) preceded target stimuli, indicating the effective rule explicitly or implicitly. The time interval between cue onset and target onset amounted to 3.000 ms (cue-target interval, CTI). Subsequent cues were presented another 3.000 ms after responses to the targets (response-cue interval, RCI). Further, additional feedback stimuli (FB) were presented during the RCI (i.e., 1.000 ms after the responses) on P-S cuing conditions. Feedback was provided by displaying the German words for “correct” (“richtig”) and “wrong” (“falsch”), respectively, in black capital letters at the center of the screen. On F-B cuing conditions, responses were followed by a 3.000 ms time interval (RCI) until the onset of hybrid C/FB stimuli. This stimulus informed, explicitly or implicitly, whether the applied set was correct or not. The onset of C/FB stimuli was followed by another interval of 3.000 ms until the onset of the subsequent target stimuli (CTI). On transition-cuing conditions, C or C/FB stimuli were composed of the German words for “repeat” (“bleiben”) and “switch” (“wechseln”), respectively, superimposed on a black rectangle. On task-cuing conditions, these stimuli consisted of the German words for “color” (“Farbe”), “shape” (“Form”) and “number” (“Zahl”). All word stimuli were of equal size (2° horizontal visual angle, font: Arial 28), and their presentation duration amounted to 200 ms. Note that the two (i.e., color, shape) or three (i.e., color, shape, number) task cues occurred about equally often (i.e., in 50% vs. 50% or in 33% vs. 33% vs. 33% of the trials, respectively) on their respective two-task or three-task experimental conditions. In contrast, “switch”-cues occurred less frequently than “repeat”-cues on transition-cuing conditions [i.e., “switch”-cues on around 25% (31 out of 120) of the trials vs. “repeat”-cues on around 75% (89 out of 120) of the trials], irrespective of the number of viable task rules on the particular experimental condition.

Participants were instructed that their task would be to match the stimulus card with one of the four key cards in accordance with the appropriate sorting rule. They were further informed that the prevailing sorting rule would change from time to time in an unpredictable manner. They were told that cue stimuli or feedback stimuli, respectively, conveyed information about the correct sorting rule. Participants were discouraged to guess whether or not task rules might have changed on the current trial. They were further instructed to prioritize response accuracy over response speed. Prior to each experimental condition, participants' understanding of the particular task at hand was ascertained by running fifteen practice trials before the corresponding 120-trial sequence was initiated.

### Experimental design

The combination of number of viable task rules (two vs. three rules), cue contingency (P-S cuing vs. F-B cuing), and cue explicitness (task cuing vs. transition cuing) provided a 2 × 2 × 2 factorial design. Participants were examined on two separate days (time lag: *M* = 6 days ± 0.9 (*SE*), range 1–13 days) since each participant had to complete all eight experimental conditions (adding to 960 trials per participant). The sequential design of the experiment was balanced across participants, as follows: (1) 50% of the participants (*N* = 8) started with all two-task conditions on the first day, and 50% of the participants (*N* = 8) started with all three-task conditions on the first day. (2) Within each day, 50% of the participants (*N* = 4) started with P-S cuing, while 50% of the participants (*N* = 4) started with F-B cuing. (3) Finally, within each day and within each cue contingency condition, the order of task cuing and transition cuing conditions was balanced across participants, with 50% of the participants (*N* = 2) starting with task cuing, and 50% of the participants (*N* = 2) starting with transition cuing. Thus, the number of viable task rules constituted the slowest-changing factor, whereas cue explicitness formed the fastest-changing factor.

### Electroencephalographic (EEG) recordings

Continuous EEG was recorded by means of a QuickAmps-72 amplifier (Brain Products, Gilching, Germany; www.brainproducts.com) and the BrainVision Recorder® Version 1.02 software (Brain Products, Gilching, Germany; www.brainproducts.com) from frontal (F7, F3, Fz, F4, F8), central (T7, C3, Cz, C4, T8), parietal (P7, P3, Pz, P4, P8), occipital (O1, O2), and mastoid (M1, M2) sites. Ag-AgCl EEG electrodes were used. They were mounted on an EasyCap (EasyCap, Herrsching-Breitbrunn, Germany; www.easycap.de). Electrode impedance was kept below 10 kΩ. All EEG electrodes were referenced to average reference. Participants were informed about non-cerebral artifacts and asked to avoid eye and limb movements as well as bucco-facial muscle activities (Picton et al., 2000). Ocular artifacts were monitored by means of bipolar pairs of electrodes positioned at the sub- and supraorbital ridges (vertical electrooculogram, vEOG) and at the external ocular canthi (horizontal electrooculogram, hEOG). The EEG and EOG channels were amplified with a band-pass of 0.01–40 Hz and digitized at 250 Hz.

Off-line analysis was performed by means of the BrainVision Analyzer® Version 2.0 software (Brain Products, Gilching, Germany; www.brainproducts.com). The contribution of ocular artifacts to the EEG was eliminated by applying independent component analysis. A digital high-pass filter was applied to the data (0.33 Hz, 24 db/oct) in order to eliminate low-frequency variations in the EEG signal which were associated with the occasional occurrence of electro-dermal artifacts. Further artifacts were removed semi-automatically [maximum allowed voltage step per sampling point: 50 μ V; maximum allowed amplitude difference: 70 μ V; lowest allowed activity (max-min, interval length 100 ms): 0.5μ V]. In a second step, the accuracy of the initially automatic artifact rejection was approved by visual inspection. By applying these rejection criteria, less than one percent of trials had to be discarded.

The EEG was then divided into epochs of 1.100 ms duration, starting 100 ms before onset of the events of interest (see below). A 100 ms pre-stimulus baseline was subtracted from the sampling points before the EEG was averaged off-line. Further, all EEG electrodes were re-referenced to the algebraic average of both mastoid electrodes, (M1 + M2)/2. However, since the resulting ERP waveforms did not differ substantially between the two reference methods, analyses and results are reported for average reference only.

### Data analysis

SPSS 13.0 served for statistical analyses (IBM, Armonk, NY; http://www-01.ibm.com/software/analytics/spss). Statistical significance level was set at *p* < 0.01.

### Behavioral data

For the eight experimental conditions, median response times (RTs) were computed for each participant, separately for set-shift trials and for first set-repetition trials (see Table 1). Comparing response latencies across these trials allows quantifying behavioral switch costs (Kiesel et al., 2010). These median RTs were subjected to a 2 × 2 × 2 × 2 analysis of variance (ANOVA) with number of viable task rules (two vs. three), cue contingency (P-S cuing vs. F-B cuing), cue explicitness (task cuing vs. transition cuing), and trial (set-shift trial vs. first set-repetition trial, Table 1) as within-subject factors. Only error-free trial sequences were selected for analysis since we aimed at eliminating confounding contributions of potential post-error slowing (Rabbitt, 1966). In addition, mean error percentages across the initial three trials of task runs were calculated (Table 1).

### EEG data analysis

ERP measures were primarily derived from stimulus-locked averages of error-less trials (which are denoted here as *COR*). Averaging was generally conducted for *C*_*x*_ and *FB*_*x:cor*_, *x* = 1 … 3, stimuli, i.e., separately for individual trials of task runs on P-S cuing conditions (Figure 1). Likewise, on F-B cuing conditions, averaging was conducted for *C*/*FB*_*y:cor*_, *y* = 2 … 3, stimuli. In addition, ERPs were analyzed in response to *C*/*FB*_1:*inc*_ stimuli on all F-B cuing conditions (which served as switch cues). Finally, ERPs evoked by *FB*_1:*inc*_ on P-S cuing conditions and those elicited by *C*/*FB*_2:*inc*_ on F-B cuing conditions were analyzed when three rules were viable and cuing was implicit (i.e., on transition cuing). Note that sufficiently large numbers of error trials for analysis were only available in these, but not in other, conditions (Table 4).

**Table 4:** **Mean (standard error) percentages of errors across conditions and trials**.

	**First trial**	**Second trial**	**Third trial**
**P-S cuing**	**(Set shift)**	**(First set repetition)**	
Task (2 tasks)	3.91 (1.09)	1.17 (0.48)	0.78 (0.61)
Task (3 tasks)	4.69 (1.31)	0.76 (0.44)	0.98 (0.47)
Transition (2 tasks)	6.35 (1.55)	2.34 (1.19)	1.36 (0.57)
Transition (3 tasks)	49.61 (3.00)	5.27 (1.09)	1.37 (0.64)
**F-B cuing**		**(Set shift)**	**(First set repetition)**
Task (2 tasks)	96.48 (2.50)	3.52 (1.24)	1.37 (0.49)
Task (3 tasks)	98.83 (0.39)	5.27 (1.50)	2.73 (0.85)
Transition (2 tasks)	96.68 (1.79)	3.13 (1.31)	3.13 (0.90)
Transition (3 tasks)	99.22 (0.35)	45.51 (2.17)	3.71 (0.96)

The table shows the initial three trials of task runs.

Mean P3a amplitudes were measured at electrode Fz in a 120 ms (±60 ms) interval around P3a peak latencies for each participant. P3a peak latencies corresponded to the largest positive deflection in individual ERP waveforms within the latency range between 340 and 440 ms following stimulus onset. P3a peak detection was performed on low-pass filtered individual ERP waveforms (12 Hz, 48 db/octave) which showed prominent P3a waves. Specifically, individual ERP waveforms evoked by *FB*_1:*cor*_ and *FB*_1:*inc*_ stimuli on three-task P-S transition cuing conditions and by *C*/*FB*_2:*cor*_ and *C*/*FB*_2:*inc*_ stimuli on three-task F-B transition cuing conditions (i.e., the 2AC trials, Table 2) served for determining individual P3a peak latencies. Averaged individual P3a peak latencies were calculated for comparisons with mean P3a amplitudes obtained from the remaining conditions which did not show prominent P3a waves. Specifically, the average latency, *av*_*P* − *S*_ = (latency *FB*_1:*cor*_ + latency *FB*_1:*inc*_)/2 on three-task P-S transition cuing conditions served as the midpoint of the 120 ms (±60 ms) latency window on the remaining P-S cuing conditions, whereas the average latency, *av*_*F* − *B*_ = (latency *C*/*FB*_2:*cor*_ + latency *C*/*FB*_2:*inc*_)/2 on three-task F-B transition cuing conditions served as the midpoint of the 120 ms (±60 ms) latency window on the remaining F-B cuing conditions. Mean P3b amplitudes were generally measured at electrode Pz in the interval between 450 ms and 700 ms following stimulus onset since the P3b did not show obvious peaks in individual ERP waveforms.

## Results

### Behavioral results

As can be seen by inspection of Table 4, response accuracy generally increased across the initial three trials of task runs (Table 1). Error proportions on these trials demonstrated strong differences between P-S cuing and F-B cuing (Figure 2) as well as between conditions involving 1AC and 2AC task-set decisions (Table 2). Participants switched rarely to the wrong task set when confronted with 1AC decisions on first trials on P-S cuing conditions, whereas F-B cuing conditions produced errors on first trials by default. As expected, set-shift trials (Table 1) provoked around 50% erroneous responses when they were associated with 2AC task-set decisions (Table 2), i.e., on transition-cuing conditions with three viable tasks, irrespective of cue contingency (i.e., on P-S cuing and on F-B cuing conditions).

Comparing RTs by means of a 2 × 2 × 2 × 2 ANOVA with number of viable tasks (two vs. three), cue contingency (P-S cuing vs. F-B cuing), cue explicitness (task cuing vs. transition cuing), and trial (set-shift trial vs. first set-repetition trial) as within-subject factors revealed a significant effect of the trial factor [*F*_(1, 15)_ = 14.94, *p* < 0.01], indicating the general occurrence of RT switch costs across experimental conditions (set-shift trials *M* = 1.109 ms; first set-repetition trials *M* = 1.009 ms), since no other main or interaction effect proved significant (all *p* > 0.02).

### ERP results

The analysis of the ERP waveforms has three main parts: (1) confirmatory evaluation of the P3b surprise hypothesis, (2) confirmatory evaluation of the P3a entropy hypothesis, and (3) exploratory evaluation of outcome valence effects.

### Evaluation of the P3b surprise hypothesis

**Figure 5:**
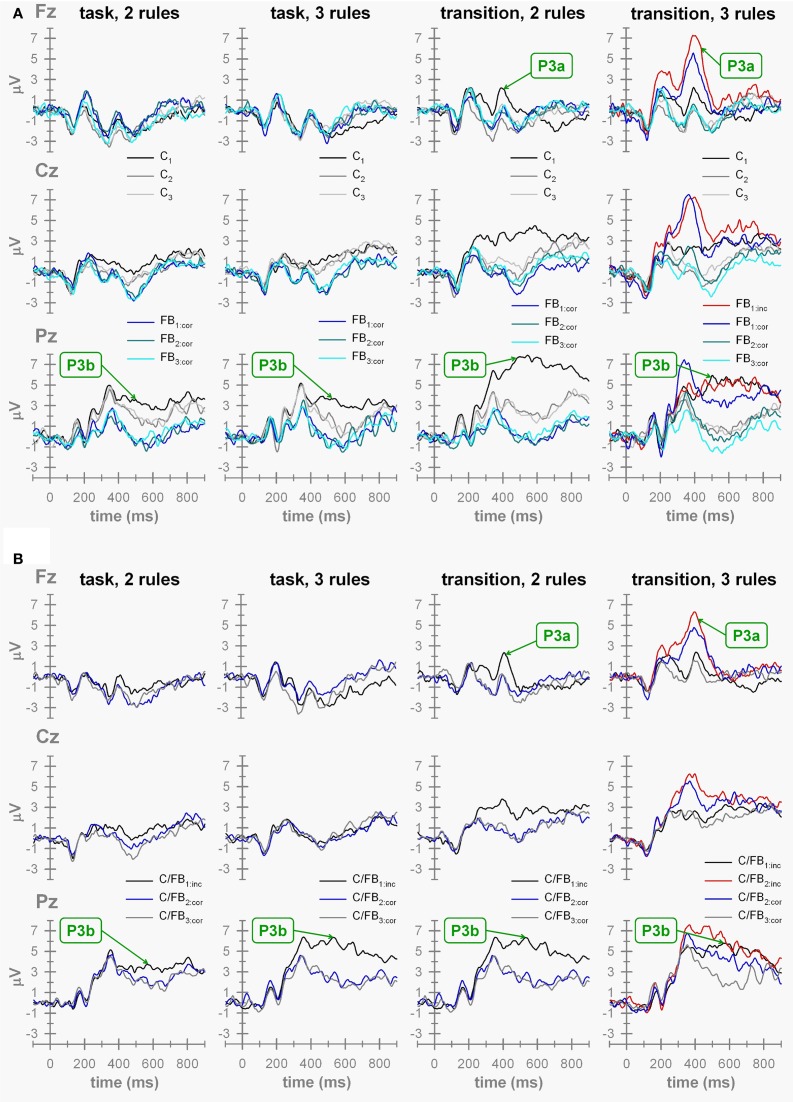
**ERP waveforms. (A)** ERP waveforms obtained on P-S cuing conditions. “task,” task-cuing conditions (two-task, three-task conditions) with C_*x*_, task cue on trial *x*{*x* = 1 … 3}; FB_*x*_:*cor*, correct feedback stimulus on trial *x*{*x* = 1 … 3}. “transition”, transition-cuing conditions (two-task, three-task conditions) with C_*x*_ = task cue on trial *x*{*x* = 1 … 3}; FB_*x*_:*cor* = correct feedback stimulus on trial *x*{*x* = 1 … 3} and FB_1:*inc*_, incorrect feedback stimulus on trial 1. **(B)** ERPs waveforms obtained on F-B cuing conditions. “task,” task-cuing conditions (two-task, three-task conditions) with C/FB_x_, hybrid cue/feedback stimulus on trial *x*{*x* = 1 … 3; “cor,” correct; “inc,” incorrect}. “transition,” transition-cuing conditions (two-task, three-task conditions) with C/FB_*x*_, hybrid cue/feedback stimulus on trial *x*{*x* = 1 … 3; “cor,” correct; “inc,” incorrect}.

Switch-related effects on cue-locked ERP waveforms can be gleaned from inspection of Figure 5 by comparing ERPs (a) evoked by *C*_1_ (switch) cues vs. those elicited by *C*_2_ and *C*_3_ (repeat) cues on P-S cuing conditions, and (b) evoked by *C*/*FB*_1:*inc*_ (switch) stimuli vs. those elicited by *C*/*FB*_2:*cor*_ and *C*/*FB*_3:*cor*_ (repeat) stimuli on F-B cuing conditions (Figure 2).

As a proxy for switch-related effects, we compared *C*_1_-locked vs. *C*_3_-locked P3b waveforms on P-S cuing conditions and *C*/*FB*_1:*inc*_-locked vs. *C*/*FB*_3:*cor*_-locked P3b waveforms on F-B cuing conditions. The resulting 2 × 2 × 2 × 2 ANOVA with number of viable tasks (two vs. three), cue contingency (P-S cuing vs. F-B cuing), cue explicitness (task cuing vs. transition cuing), and trial (first trial vs. third trial) as within-subject factors showed a significant explicitness by trial interaction, *F*_(1, 15)_ = 29.01, *p* < 0.01, indicating more pronounced P3b amplitude switch-related effects on transition cuing conditions compared to task cuing conditions. Separate analyses for the two levels of cue explicitness revealed that P3b amplitudes showed significant switch-related effects on both conditions, *F*_(1, 15)_ = 25.33, *p* < 0.01 (task-cuing condition), *F*_(1, 15)_ = 166.51, *p* < 0.01 (transition-cuing condition). Thus, we found evidence for switch-related effects on parietal P3b amplitudes as they are consistently reported in the task-switching literature (Karayanidis et al., 2010). Over and above this often replicated finding, the switch-related effects on parietal P3b amplitudes were modulated by cuing method such that more pronounced switch-related effects occurred on transition-cuing conditions.

### Evaluation of the P3a entropy hypothesis

**Figure 6:**
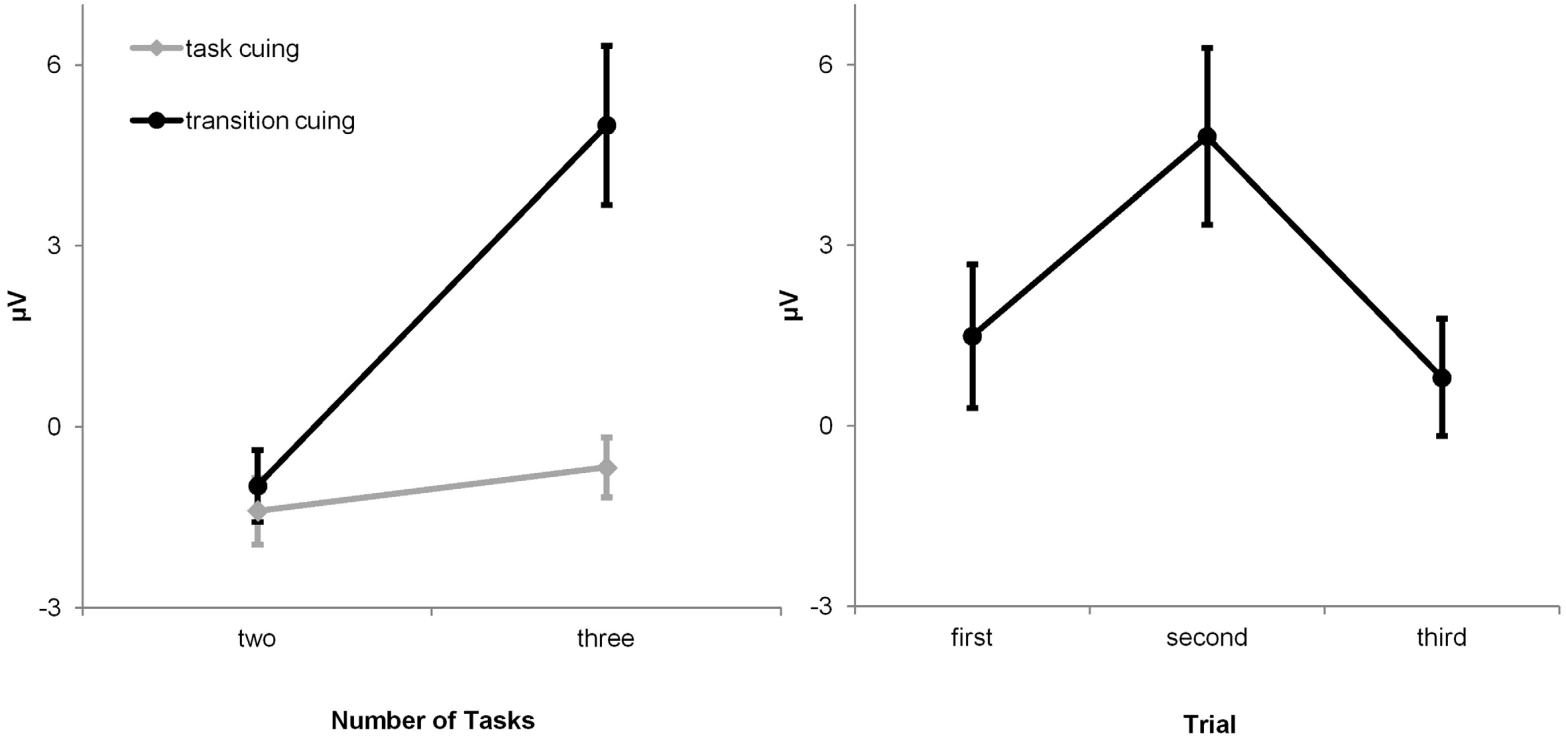
**Mean (standard error) P3a amplitudes at Fz. Left panel:** The interaction between cue explicitness and number of tasks reveals that P3a amplitudes vary as a function of post-switch outcome uncertainty which is high on three-task transition-cuing conditions and low on all remaining conditions. **Right panel:** P3a amplitudes follow the entropy function over outcome events across the initial three trials of task runs on the uncertain F-B cuing condition.

Two ANOVAs were conducted to evaluate the hypothesis. The first ANOVA targeted the effects of post-switch outcome uncertainty (i.e., *FB*_1_-locked P3a amplitudes on P-S cuing conditions and *C*/*FB*_2_-locked P3a amplitudes on F-B cuing conditions) across all experimental conditions (Figure 5). Inspection of the ERP waveforms reveals that pronounced P3a waveforms were solely evoked by *FB*_1_- and by *C*/*FB*_2_-feedback stimuli on three-task transition-cuing conditions, i.e., when the correctness of task-set decisions was unpredictable. This impression was corroborated by the results of the 2 × 2 × 2 ANOVA with number of viable tasks (two vs. three), cue contingency (P-S cuing vs. F-B cuing) and cue explicitness (task cuing vs. transition cuing) as within-subject factors. This ANOVA showed significant effects of number of tasks, *F*_(1, 15)_ = 27.89, *p* < 0.01, cue explicitness, *F*_(1, 15)_ = 45.01, *p* < 0.01, as well as of the number of tasks by cue explicitness interaction, *F*_(1, 15)_ = 24.92, *p* < 0.01, with all remaining *F*-values < 1. The interaction between number of viable tasks and cue explicitness is illustrated in Figure 6 (left panel). Separate ANOVAs were performed on each number of tasks condition to further parse the two-way interaction. These analyses revealed that P3a amplitudes showed significant cue explicitness effects on three-task conditions, *F*_(1, 15)_ = 37.13, *p* < 0.01 (task cuing: *M* = −0.98 μV, transition cuing: *M* = 5.00 μV), but not on two-task conditions, *F*_(1, 15)_ = 5.83, *p* > 0.01 (task cuing: *M* = −1.40 μV, transition cuing: *M* = −0.60 μV). Thus, post-switch outcome events evoked enhanced P3a amplitudes specifically when three viable tasks were combined with transition cues.

The second ANOVA was performed as a direct test for the P3a entropy hypothesis. Inspection of Table 4 reveals that negative-outcome probabilities across task runs on the three-task F-B transition cuing condition approached the *P* = 1.0, *P* = 0.5, and *P* = 0.0 values that were predicted for ideal performers (Table 3). Inspection of Figure 3 reveals that the P3a entropy hypothesis predicts enhanced P3a amplitudes elicited by *P* = 0.5 outcomes in comparison to both, *P* = 1.0 and *P* = 0.0 outcomes, which should indistinguishably evoke less prominent P3a waveforms. The omnibus ANOVA on P3a amplitudes evoked by *C*/*FB*_2_ (with *P*_*w*_ → 0.5), *C*/*FB*_1_ (with *P*_*w*_ → 1.0), and *C*/*FB*_3_ (with *P*_*w*_ → 0.0), outcomes, *F*_(1, 15)_ = 21.39, *p* < 0.01, was followed by planned Helmert contrasts, i.e., (1) *C*/*FB*_2_ *vs*. (*C/FB*_1_ + *C/FB*_3_)/2, *F*_(1, 15)_ = 35.72, *p* < 0.01, and (2) *C*/*FB*_1_ *vs. C/FB*_3_, *F*_(1, 15)_ = 1.20, *p* > 0.01, respectively. These results are shown in Figure 6 (right panel), and they reveal that P3a amplitudes follow the predicted entropy pattern. Taken together, both ANOVAs devoted to the evaluation of the P3a entropy hypothesis showed that amplitude variability in P3a was related to outcome uncertainty, and more specifically, to the entropy over switch outcomes.

### Analysis of outcome valence effects

An exploratory comparison of ERP waveforms evoked by (roughly equiprobable, cf. Table 4) positive and negative outcomes was possible by comparing *FB*_1:*cor*_ and *C*/*FB*_2:*cor*_ against *FB*_1:*inc*_ and *C*/*FB*_2:*inc*_, respectively. The resulting 2 × 2 ANOVAs with cue contingency (P-S cuing vs. F-B cuing) and outcome valence (positive vs. negative) as within-subject factors showed significant valence effects, *F*_(1, 15)_ = 14.68, *p* < 0.01, for the P3b, but not for the P3a, *F*_(1, 15)_ = 5.83, *p* > 0.01, indicating enhanced P3b, but not P3a, amplitudes evoked by negative outcomes compared to positive outcomes. Implications of this finding will be considered in the discussion section.

## Discussion

This study explored relationships between various aspects of uncertainty and neuronal correlates of cognitive control in several modifications of a cued task-switching paradigm. Subjects switched between two or three task sets; contextual cues were either (explicit) task cues or (implicit) transition cues, and contextual cuing was either prospectively-signaled or feedback-based in WCST-like task-switching paradigms (Barceló, 2003). The manipulated variables of our experimental design exerted negligible influence on behavioral switch costs, as evidenced by their general and indistinguishable presence on all eight experimental conditions. There were two separable ERP components which could be easily distinguished by their topography. More importantly, they were dissociated by their antecedent conditions. The switch-related parietal P3b was commonly evoked by switch-cues on all experimental conditions, with the switch-related variability in P3b amplitude being more pronounced on transition cuing. Further, the P3b distinguished negative and positive outcomes. A frontal P3a was evoked by feedback stimuli, and it distinguished certain from uncertain task switching such that solely uncertain switch outcomes elicited a prominent P3a, irrespective of their valence. Each of these ERP components is discussed in turn.

### Switch-related amplitude variability in P3b

The switch-related parietal P3b has been present in many different task-switching experiments (see Karayanidis et al., 2010 for review), so that it has been difficult to assign any specific functional role to it. As one possibility, the switch-related P3b may index the updating of “attentional set” in our study, i.e., the updating of the relevant features of those stimuli which later determine action (Rushworth et al., 2005). However, since the switch-related P3b has so often been observed in a diversity of task-switching experiments, we prefer to interpret its occurrence not as an index of switching between attentional sets, but rather as indicating being unprepared to update (and, conversely, being prepared to repeat) task sets, irrespective of the nature of the particular sets concerned.

This idea was dubbed P3b surprise hypothesis in the introduction to this article; it has the advantage of being generally applicable to any kind of study. In fact, the historical roots of the P3b surprise hypothesis (Donchin, 1981) lie in oddball paradigms (e.g., Squires et al., 1976), and it is one of the major functional models of variability in parietal P3b amplitude (Kolossa et al., 2013). The predictions of the surprise model are clear in terms of how absolute switch probability should affect switch-related variability in P3b amplitude. As revealed by inspection of Figure 3, surprise shows an asymmetrical course over binary probability distributions, with maximal surprise when *P* → 0.0 and minimal surprise when *P* → 1.0. Thus, surprise over switches (*P* = 0.25) exceeds surprise over repetitions (*P* = 0.75) throughout all conditions of our experiment, thereby offering an explanation for the switch-related amplitude variability in P3b. In contrast to this, the switch-related amplitude variability in P3b cannot be accommodated to entropy over switches and repetitions, due to the symmetrical course of entropy over binary probability distributions. It is further of importance to note that a P3b occurred in response to all switch cues, irrespective of the level switch uncertainty.

It should be kept in mind, however, that our study does not directly pertain to the P3b surprise model since we did not manipulate absolute switch probability. How is task-switching affected by switch probability? In one of the few studies of this type, Monsell and Mizon (2006; Experiment 4) manipulated switch probability (*P* = 0.25, *P* = 0.5, or *P* = 0.75) and showed that behavioral switch costs decreased as switches became more frequent. Further, Nessler et al. (2012) recently found that switch-related amplitude variability in P3b disappeared completely when switches and repetitions were equiprobable, *and* when the sequential succession of switches and repetitions was highly predictable. With regard to this, equiprobable switches and repetitions are, in and of itself, not sufficient for eliminating surprise over task-set switches (Barceló et al., 2006). Consider the possible succession of task-set switches (*s*) and task repetitions (*r*) over three consecutive trials (*n-2*, *n-1*, *n*), given switch probability *P* = 0.5. The succession of two task repetitions, i.e. *r*_(*n* − 1)_*r*_(*n*)_, is equivalent to the succession of a particular task (with tasks denoted *a* or *b*), *a*_(*n* − 2)_*a*_(*n* − 1)_*a*_(*n*)_, and a repetition after a switch, *s*_(*n* − 1)_*r*_(*n*)_, equals the task sequence, *b*_(*n* − 2)_*a*_(*n* − 1)_*a*_(*n*)_. Thus, if a cue on (*n*) is the eliciting event, the short-term task probability for task *a* on the preceding two trials, (*n* − 2) and (*n* − 1), equals *P*(*a*) = 0.75. However, the succession of two task-set switches, i.e. *s*_(*n* − 1)_*s*_(*n*)_, is equivalent to **a**_(*n* − 2)_*b*_(*n* − 1)_*a*_(*n*)_, and a switch after a repetition, *r*_(*n* − 1)_*s*_(*n*)_, equals the task sequence, *b*_(*n* − 2)_*b*_(*n* − 1)_*a*_(*n*)_. Thus, if a cue on (*n*) is the eliciting event, the short-term task probability for task *a* on the preceding two trials (*n* − 2) and (*n* − 1) equals *P*(*a*) = 0.25. The confound between switch probability (*P* = 0.5) and short-term task probability (*P*(*a*_(*n* − 2)(*n* − 1)_/*r*_(*n*)_) = 0.75, *P*(*a*_(*n* − 2)(*n* − 1)_/*s*_(*n*)_) = 0.25) will affect amplitude variability in P3b (Squires et al., 1976; Kolossa et al., 2013).

The switch-related variability in P3b amplitude was more pronounced on transition cuing. This modulation may be related to the higher memory load which is associated with transition cues in comparison to task cues (Schneider and Logan, 2007). However, the P3b surprise hypothesis offers an alternative explanation for this result. Whereas switch probability was held constant across transition-cuing and task-cuing conditions, stimulus probability distinguished between the two conditions. Specifically, task cuing implies physically identical switch cues and repeat cues, their sole difference lying in their serial position within task runs (switch cues initiate new task runs, e.g., … *bb*a … (switch cue underlined), whereas repeat cues do not, … *a*aa … (repeat cues underlined)). This is different if one considers stimuli on transition-cuing conditions, with physically different switch cues and repeat cues (labeled *switch* and *repeat*), e.g., … *repeat repeat* switch … (switch cue underlined). Given switch probabilities of *P* = 0.25, transition switch cues occurred less frequently than transition repeat cues. Thus, the sensory surprise over switch cues on transition-cuing conditions exceeds the sensory surprise over switch cues on task-cuing conditions, and it is well known that sensory surprise contributes to the switch-related variability in P3b amplitude (Nicholson et al., 2006; Jost et al., 2008; Periáñez and Barceló, 2009.

The use of Karayanidis et al. (2009) “switch-to” (equivalent to explicit task cues) vs. “switch away” (equivalent to transition cues) cuing may help to circumvent the sensory surprise confound when comparisons between switches under task cuing and switches under transition cuing are under scrutiny. In this paradigm, task cuing relies on mapping tasks with particular spatial positions. “Switch-to” cuing is mapped to the spatial position of one particular task (thereby unambiguously defining a task-set), whereas two viable tasks are cued by indicating spatial positions with overlapping task mappings in “switch away” cuing (thereby ambiguously defining two task sets).

### Feedback-related amplitude variability in P3a

One of the basic findings of the study was that the frontal P3a was evoked by feedback stimuli rather than by cue stimuli. The appearance of a feedback-locked P3a is a common denominator of prospectively-signaled and feedback-based task switching. These data are in agreement with earlier work in this area. It has repeatedly been reported that *C/FB*-events in WCST-like task-switching paradigms evoke prominent P3a waveforms (Barceló et al. (2002); Barceló, 2003; Kopp et al., 2005, 2006). However, Cunillera et al. (2012) recently reported that switch cues (*C*_1_-cues) and feedback events (*FB*_1_-events) evoked prominent P3a waveforms of similar amplitudes on a prospectively-signaled, three-task transition task-switching paradigm, seemingly contradicting our finding of specifically outcome-evoked amplitude variability in P3a. While the data converge with regard to the P3a evoked by *FB*_1_-events, they seem to diverge with regard to the P3a evoked by *C*_1_-cues. The contradictory pattern of results can be explained by the instrumental character of *C*_1_-cues in Cunillera et al.'s (2012) study in which they signaled the need for task-set updating, akin to the role of *FB*_1:*INC*_-events in both studies. Note that task-set switches are only one prerequisite for task-set updating; the need for task-set updating also emerges when an error occurred on the preceding trial. In contrast, *C*_1_-cues in our study indicated task-set switches, irrespective of foregone errors, as detailed above (cf. Figure 2). Thus, *C*_1_-cues provided information about task-set switches and/or foregone errors in Cunillera et al.'s study (i.e., they served as hybrid *C/FB*-stimuli). In contrast, *C*_1_-cues solely informed about task-set switches, irrespective of foregone errors, in the current study (i.e., they served as pure *C*-stimuli).

There were some differences between the experimental conditions which, apparently, exerted only minor effects on amplitude variability in P3a. First, the duration of the response-stimulus intervals differed between prospectively-signaled (one second) and feedback-based (three seconds) cuing conditions (cf. Figure 2). Second, the semantic framing of feedback-stimuli differed between prospectively-signaled (*wrong*, *right*) and feedback-based (*switch*, *stay*) cuing conditions. Future studies should keep these variables constant, or they should address their role by manipulating them systematically, despite the current evidence of their seemingly negligible role for amplitude variability in P3a.

The frontal P3a which was evoked by feedback stimuli on both cuing-contingency conditions distinguished certain from uncertain task switching such that solely uncertain switch outcomes elicited a prominent P3a. Specifically, the entropy over switch outcomes modulated the frontal P3a, such that uncertain switch outcomes (*P*_*w*_ = 0.5) elicited enhanced P3a amplitudes in comparison to certain switch outcomes (i.e., *P*_*w*_ = 1.0 and *P*_*w*_ = 0.0). Importantly, the P3a data thereby lend support to the P3a entropy hypothesis according to which modulations of P3a amplitudes are generally related to the entropy which is conveyed by eliciting stimuli (Barceló et al., 2006).

Neither the memory load hypothesis (Schneider and Logan, 2007) nor the contingency hypothesis (Kopp and Wessel, 2011) received support from the P3a results obtained in this study. Inspection of Figure 5 reveals that transition switch cues, but not task switch cues, evoked small enhancements of frontal P3a waves. However, the main effect of cue explicitness in the corresponding ANOVA is difficult to interpret, given the significant interaction between the number of tasks and cue explicitness. Further, the non-significance of cue explicitness effects on two-task conditions suggests that cue explicitness exerts rather marginal effects on P3a amplitude variability, and that they may be difficult to obtain (but see West et al., 2011). The contingency hypothesis (Kopp and Wessel, 2011) was incorrect insofar as it held that P3a amplitude enhancements should be restricted to *C/FB*-stimuli on feedback-based task switching. This prediction was clearly disconfirmed, since *FB*-stimuli on prospectively-signaled task switching evoked P3a waves of similar amplitude. This result indicates that the unique combination of task information and performance feedback by *C/FB*-stimuli is *not* necessary for obtaining a prominent P3a in response to switch outcome events. In conclusion, the main P3a findings of this study are that amplitude variability in P3a is (a) feedback-locked, and (b) related to the uncertainty of switch outcomes.

### A note on the effects of outcome valence on ERP waveforms

Amplitude variability in the feedback-related negativity (FRN) is a frequent subject in ERP studies of outcome processing and feedback-guided learning (see San Martín, 2012, for review). The FRN is a frontally distributed negative ERP component (around 250 ms after outcome presentation) which tends to be larger for negative than for positive outcomes (i.e., the outcome valence effect on amplitude variability in FRN). Holroyd and Coles (2002) proposed that the FRN is a scalp-recorded index of a neuronal system for reinforcement learning (RL; Sutton and Barto, 1998). However, inspection of Figure 5 reveals that outcome valence (which could only be analyzed in the two uncertain switching conditions) did obviously not evoke FRN-like amplitude variability in the current study.

In this study, a P3b, rather than a FRN, outcome valence effect was observed (with a larger P3b elicited by negative outcomes). San Martín (2012) concluded that there are inconsistent findings in the literature regarding the relationship between outcome valence and amplitude variability in P3b. We suggest that the effect of outcome valence on the P3b might result from another variable, since negative, but not positive, outcomes signal the need for additional task-set switches (see Table 2). Thus, negative outcomes, but not positive outcomes, serve as switch cues, and the apparent outcome valence effect may simply be another instance of switch-related amplitude variability in P3b.

### Toward a theoretical integration of uncertainty and cognitive control

The results of our study imply that switch-related P3b and P3a are dissociable, with regard to their scalp topography and to their antecedent conditions (P3b: cue-locked and modified by switch surprise, irrespective of switch entropy; P3a: feedback-locked and modified by switch entropy). These ERP data await comprehensive theoretical treatment, which is outlined below.

The theoretical fundament is provided by Sokolov's (1966) entropy model of the orienting response (OR), which analysed the dependence of the OR (a) on the statistical properties of the signals and (b) on their information content (see also Velden, 1974). Let *e*_*t*_ denote an event *e* at time *t*, *P*(*e*_*t*_/*S*_*j*_) be the probability of *e* at *t* in case of one of several hypothetical states *S*_*j*_{*with j* = 1,…, *n*}, *P*(*S*_*j*_/*e*_*t*_) be the posterior probability of a particular state *S*_*j*_ given *e* at *t*, and *P*(*S*_*j*_) be the prior probability of the state *S*_*j*_. Then, according to Bayes' theorem

(9) $$P(S_{j}/e_{t})=(P(e_{t}/S_{j})\times P(S_{j}))/P(e_{t}).$$

Thus, Sokolov's model is first of all a Bayesian model (Knill and Pouget, 2004; Doya et al., 2007; Friston, 2010; Vilares and Körding, 2011; Penny, 2012) of the OR. Further, the degree of uncertainty is determined by the entropy over the probability distribution of posterior probabilities of all hypothetical states, i.e.,

(10) $$H(S_{j=1\ldots n}/e_{t})=-(P(S_{1}/e_{t})\log_{2}P(S_{1}/e_{t})+\ldots +P(S_{n}/e_{t})\log_{2}P(S_{n}/e_{t})).$$

According to Sokolov (1966), the OR arises when the uncertainty of the situation, depending on the number of hypothetical states, reaches the threshold value, i.e., when *H*(*S*_*j* = 1…*n*_/*e*_*t*_) > threshold, and the OR lasts until the uncertainty is eliminated and *H*(*S*_*j* = 1… *n*_/*e*_*t*_) < threshold^1^. Further, Sokolov introduced the term “enquiries” to describe *conditioned orienting reactions* to information-carrying signals, i.e., a “selective concentration of attention at definite moments in time” (Sokolov, 1966, p.353). Conditioned orienting reactions are not evoked by the level of entropy as such (which is nevertheless necessarily required), “but its change anticipated at a given moment in time” (Sokolov, 1966, p.353).

Sokolov's (1966) entropy model of conditioned orienting reactions can be applied directly to uncertain task switching. Here, uncertain switch cues induce entropy, whereas uncertain switch outcomes eliminate entropy. Thus, viewed from the perspective of Sokolov's (1966) model, the elicitation of a P3a by uncertain switch outcomes is equivalent to a conditioned orienting reaction, an attentional focusing at that moment in time when an information-carrying (entropy-eliminating) signal was anticipated. In that context, it is of interest that the P3a component of the ERP has often been considered as indicating the brain's OR (e.g., Friedman et al., 2001; Barceló et al. (2002); Nieuwenhuis et al., 2011). Further, our analysis is very similar to Barcelü et al.'s (2006) P3a entropy hypothesis. However, a subtle difference between these two models lies in the fact that our model does not relate the P3a in uncertain task-switching paradigms to stimuli which *convey* entropy (i.e., uncertain switch cues), but rather to stimuli which *eliminate* entropy (i.e., uncertain switch outcomes).

**Figure 7:**
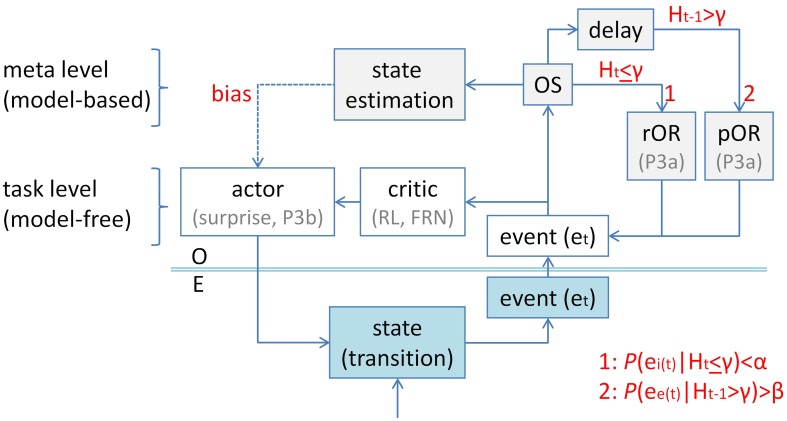
**An application of Sokolov's (1966) entropy model of the orienting response to task switching**. An actor-critic architecture (Sutton and Barto, 1998) is assumed at the task level (white frames; with P3b being related to the surprise over switches, FRN being related to negative outcomes). Perception of event *e* at time *t* (*e*_*t*_) activates processing at the task level, and it initiates processing in the multi-faceted orienting system (OS; light gray frames), which leads under two different conditions to an orienting response (OR): (1) If *e*_*t*_ is entropy-inducing (*e*_*i*_) and unexpected, i.e., *P*(*e*_*i*(*t*)_) < α (with α → 0), under a non-entropic measure at time *t*, i.e., *H*_*t*_ < γ. (2) If *e*_*t*_ is entropy-eliminating (*e*_*e*_) and expected, i.e., *P*(*e*_*e*(*t*)_) > β (with β → 1), under an entropic measure at delayed time *t*-1, i.e., *H*_*t* − 1_ > γ. In case (1), a reactive orienting response (rOR) is evoked, whereas a proactive orienting response (pOR) is released in case (2). Further, the OS serves as a switch from task control at the task level to task control at the meta level. One of the functions of meta-level processing is to estimate the current state of the environment from a number of hypothetical states, i.e., to dynamically model state transitions which occur in the environment (turquoise frames). Hence, the meta level is considered being “model-based,” whereas the task level is considered being “model-free.” Computationally, state estimation is equivalent to solving inverse problems. Once a state has been estimated, the meta level of task control is able to bias the actor toward adequate stimulus-response mappings. In its application to task switching, task control is exerted at the task level as long as *H*_*t*_ < γ (e.g., during task runs). When *P*(*e*_*i*(*t*)_) < α is not met, entropy-inducing switch cues fail to evoke a rOR. However, entropy-eliminating switch outcomes are expected to occur at time *t*, i.e., *P*(*e*_*e*(*t*)_) > β, under the delayed entropic measure, *H*_*t* − 1_ > γ, which is a consequence of the occurrence of entropy-inducing switch cues at time *t*-1. Therefore, the uncertainty-related P3a qualifies as a pOR (also labeled conditioned orienting reaction, cf. Sokolov, 1966). O, organism; E, environment; RL, reinforcement learning; FRN, feedback-related negativity.

Figure 7 shows a possible application of Sokolov's (1966) entropy model in the context of a hierarchical model of task control (Mayr et al., 2013), which specifies task-level and meta-level components. As an initial approximation, a simple actor-critic architecture (Sutton and Barto, 1998) is assumed at the task level (with P3b being related to the surprise over switches, FRN being related to negative outcomes). Perception of event *e* at time *t* (*e*_*t*_) activates processing at the task level, and it simultaneously initiates processing in the multi-faceted orienting system (OS), which leads under two different conditions to an OR: (1) If *e*_*t*_ is entropy-inducing (*e*_*i*_) and unexpected, i.e., *P*(*e*_*i*(*t*)_) < α (with α → 0), under a non-entropic measure at time *t*, i.e., *H*_*t*_ ≤ γ. (2) If *e*_*t*_ is entropy-eliminating (*e*_*e*_) and expected, i.e., *P*(*e*_*e*(*t*)_) > β (with β → 1), under an entropic measure at delayed time *t*-1, i.e., *H*_*t* − 1_ > γ. In case (1), a reactive orienting response (rOR) is evoked, whereas a proactive orienting response (pOR) is released in case (2). rORs and pORs may activate ORs by partly different neuronal mechanisms. Their final common pathway involves “selective concentration of attention at definite moments in time” (Sokolov, 1966, p.353), applied to *e*_*t*_, and measurable as P3a.

Further, the OS serves as a switch from task control at the task level to task control at the meta level (Nelson and Narens, 1990; Dosenbach et al., 2008; Tsujimoto et al., 2011; Fleming and Dolan, 2012; Kepecs and Mainen, 2012; Petersen and Posner, 2012; Yeung and Summerfield, 2012). One of the functions of meta-level processing is to estimate the current state of the environment from a number of hypothetical states, i.e., to dynamically model state transitions which occur in the environment. Hence, the meta level is considered being “model-based,” whereas the task level is considered being “model-free” (Johnson and Donchin, 1982; Daw et al., 2005; Behrens et al., 2007; Gläscher et al., 2010; Summerfield et al., 2011; Pearson and Platt, 2012). Computationally, state estimation is equivalent to solving inverse problems (Dayan et al., 1995; Friston, 2010; Kopp, 2012), putatively by Bayesian probability inversion (Sokolov, 1966; Kopp, 2008). Once a state has been estimated, the meta level of task control is able to bias the actor toward adequate stimulus-response mappings (Nelson and Narens, 1990).

In its application to task switching, task control is exerted at the task level as long as *H*_*t*_ ≤ γ (e.g., during task runs). When *P*(*e*_*i*(*t*)_) < α is not met, entropy-inducing switch cues fail to evoke a rOR. However, entropy-eliminating switch outcomes are expected to occur at time t, i.e., *P*(*e*_*e*(*t*)_) > β, under the delayed entropic measure, *H*_*t* − 1_ > γ, which is a consequence of the occurrence of entropy-inducing switch cues at time *t*-1. Therefore, the uncertainty-related P3a qualifies as a pOR (also labeled conditioned orienting reaction, cf. Sokolov, 1966).

Shimamura (2008) mapped task-level and meta-level processing to distinct cortical regions. Specifically, he assigned task-level control to areas within the posterior cortex (PC), whereas meta-level control should be associated with areas within the prefrontal cortex (PFC). Meta-level cognitive processes, placed at the top of the cognitive hierarchy (Kopp, 2012), are expected to be supported by the anterior PFC, a region being a possible top level node of task control because progressively rostral PFC regions support progressively higher levels of the cognitive hierarchy (Koechlin et al., 2003; Badre, 2008; Badre et al., 2009, 2010).

The frontopolar cortex (FPC) is the most anterior part of the PFC. An understanding of FPC cognitive functions remains elusive, yet the FPC has been implicated in several cognitive functions, such as prospective memory (Simons et al., 2006), gating external and internal influences on cognition (Burgess et al., 2007), establishing task sets (Sakai, 2008), decision making (Soon et al., 2008) and evaluating outcomes of decisions (Ramnani et al., 2004; Boorman et al., 2009). Tsujimoto et al. (2010) reported single-cell activity in FPC, demonstrating that some FPC neurons encoded decisions when feedback approached, thereby suggesting a role of FPC in evaluating decisions. According to Fleming and co-workers, individual differences in structure (Fleming et al., 2010) and function (Fleming et al., 2012) of the right rostral PFC correlated with the accuracy of confidence reports in perceptual decision making paradigms. Fleming et al. suggested that this region of the anterior PFC re-represents decision uncertainty, to facilitate reportable confidence in task performance. Here we propose, albeit speculatively, that neuronal activity in the FPC contributes to amplitude variability in uncertainty-related P3a which in turn is associated with pOR-supported elimination of entropy over hypothetical states, as described above.

## Conclusion

P3b and P3a waveforms represent separate electrophysiological markers of dissociable aspects of cognitive control in task-switching paradigms. Whereas parietal P3b waveforms are related to surprise over switches at the task level, frontally distributed P3a waveforms seem to be specifically related to the processing of uncertain switch outcomes at the meta level (functionally described as *proactive orienting responses*). P3a activities may computationally be related to the formation of inverse models of dynamic environments, as originally described by Sokolov (1966). One implication of the current research is the hypothesis that brain lesions affecting the OS lead to deficient contextual adjustment, a pervasive behavioral disturbance which represents one of the hallmarks of executive dysfunctions (Kopp, 2012).

### Conflict of interest statement

The authors declare that the research was conducted in the absence of any commercial or financial relationships that could be construed as a potential conflict of interest.

## Footnotes

^1^Sokolov (1966) used the labels *K*_*j*_ and *A*_*i*_ rather than *e*_*t*_ and *S*_*j*_, respectively.
